# Feminizing *Wolbachia* influence microbiota composition in the terrestrial isopod *Armadillidium vulgare*

**DOI:** 10.1038/s41598-018-25450-4

**Published:** 2018-05-03

**Authors:** Jessica Dittmer, Didier Bouchon

**Affiliations:** 1 Université de Poitiers, UMR CNRS 7267, Ecologie et Biologie des Interactions, équipe Ecologie Evolution Symbiose, 5 rue Albert Turpin, 86073 Poitiers, France; 2 Present Address: Dipartimento di Biologia e Biotecnologie, Università degli Studi di Pavia, Via Ferrata 9, 27100 Pavia, Italy

## Abstract

*Wolbachia* are widespread heritable endosymbionts of arthropods notorious for their profound effects on host fitness as well as for providing protection against viruses and eukaryotic parasites, indicating that they can interact with other microorganisms sharing the same host environment. Using the terrestrial isopod crustacean *Armadillidium vulgare*, its highly diverse microbiota (>200 bacterial genera) and its three feminizing *Wolbachia* strains (*w*VulC, *w*VulM, *w*VulP) as a model system, the present study demonstrates that *Wolbachia* can even influence the composition of a diverse bacterial community under both laboratory and natural conditions. While host origin is the major determinant of the taxonomic composition of the microbiota in *A. vulgare*, *Wolbachia* infection affected both the presence and, more importantly, the abundance of many bacterial taxa within each host population, possibly due to competitive interactions. Moreover, different *Wolbachia* strains had different impacts on microbiota composition. As such, infection with *w*VulC affected a higher number of taxa than infection with *w*VulM, possibly due to intrinsic differences in virulence and titer between these two strains. In conclusion, this study shows that heritable endosymbionts such as *Wolbachia* can act as biotic factors shaping the microbiota of arthropods, with as yet unknown consequences on host fitness.

## Introduction

Heritable symbiotic bacteria are essential drivers of arthropod ecology and evolution. This is exemplified by the many species harbouring obligate or facultative vertically transmitted endosymbionts and the diversity of symbiont effects on host fitness^1,2^. These effects can be beneficial, e.g. providing essential nutrients lacking from the host’s diet or defence against natural enemies^3–11^ or parasitic, including reproductive parasitism^12–14^.

Bacteria of the genus *Wolbachia* are probably the most widespread heritable bacterial endosymbionts, infecting a wide range of arthropods (up to 65% of insect species are estimated to be infected^15,16^) and filarial nematodes^17^. Despite several cases of mutualism or dependence^18–23^, most arthropod-infecting *Wolbachia* are reproductive parasites: Being maternally transmitted, they manipulate their hosts’ reproduction in various ways (i.e. cytoplasmic incompatibility (CI), parthenogenesis, male-killing or the feminization of genetic males) to promote their own vertical transmission^24–28^. Although far from being the only bacteria inducing these phenotypes, *Wolbachia* are the most frequently encountered reproductive manipulators and cause the largest spectrum of reproductive phenotypes^12,15,29^.

In addition to our growing understanding of the diversity of symbiont-mediated effects on hosts, the focus of symbiosis research has recently broadened from the study of binary host-symbiont interactions to a more holistic view of a host and its associated microbial community^30–34^. From this perspective, symbioses are shaped by highly dynamic multipartite interactions, not only between the host and its symbionts but also between the different members of the symbiotic community^35–39^. The latter may be direct interactions, e.g. through competition for resources or space within the shared host^35,39–41^ or by promoting the evolution of cooperation or dependence between different bacteria^36,42–44^. Alternatively, particular taxa could provoke a host immune response, which in turn might affect the microbiota as a whole, with potential consequences for organismal function. Indeed, studies of the relatively simple gut microbiota of *Drosophila melanogaster* have revealed (i) the importance of certain commensal bacteria for larval growth and optimal nutrient metabolism as well as (ii) a fine-tuned equilibrium between the host’s innate immune response and the gut microbiota to maintain homeostasis and prevent the proliferation of bacterial pathogens^45–49^. This leads us to ask whether heritable intracellular bacterial symbionts such as *Wolbachia*, which can reach high titers in host tissues and have profound impacts on host fitness, may also interact with the rest of the bacterial community present in the same host. This is all the more relevant since, due to its wide arthropod host range, *Wolbachia* is a dominant member of the microbiota in many insects, including species of agricultural or medical importance such as whiteflies or mosquitoes^50–55^.

In the context of *Wolbachia* symbioses in arthropods, numerous studies have demonstrated a *Wolbachia*-mediated protection against various viruses and *Plasmodium* parasites in naturally infected *Drosophila* as well as in transfected mosquito species^56–62^. This protective phenomenon indicates that *Wolbachia* can indeed interact with other microorganisms in the same host environment, either indirectly via the induction of a general immune response, or via competition for resources and space. In contrast, relatively little is known regarding interactions between *Wolbachia* and other bacteria, except for several binary interactions with other highly abundant symbionts. Hence, male-killing *Spiroplasma* have been shown to negatively affect *Wolbachia* titers in *D. melanogaster*^40^ and recent studies have demonstrated a mutual competitive exclusion between *Wolbachia* and *Asaia* in the reproductive organs of *Anopheles* and *Aedes* mosquitoes, effectively inhibiting vertical transmission of the respective other symbiont^63,64^. However, *Wolbachia*’s influence on a larger commensal bacterial community has been investigated only recently in laboratory lines of *D. melanogaster*^65,66^, yielding conflicting results: While *Wolbachia* infection resulted in an overall decrease in taxonomic richness of the *Drosophila* gut microbiota, along with an increased abundance of the two bacterial families Leuconostocaceae (Firmicutes, Bacilli) and Acetobacteraceae (Alphaproteobacteria) in Ye *et al*.^66^, Simhadri *et al*.^65^ instead observed significantly reduced titers of Acetobacteraceae, notably *Acetobacter pasteurianus*. These results indicate that *Wolbachia*-microbiota interactions may be complex and dependent on both host genotype and *Wolbachia* strain.

The objective of the present study was to investigate the impact of *Wolbachia* on a diverse symbiotic bacterial community under both laboratory and natural conditions. To achieve this, we used the terrestrial isopod *Armadillidium vulgare* and its association with feminizing *Wolbachia* as a model system for several reasons: First, three different feminizing *Wolbachia* strains (*w*VulC, *w*VulM, *w*VulP) establish stable single-infections in this host^67–70^, allowing us to compare the impact of different *Wolbachia* strains without having to account for additional interactions between the different *Wolbachia* strains themselves. Second, our recent quantitative study revealed *Wolbachia* strain-specific tissue distribution patterns in this species^71^, possibly reflecting different co-evolutionary histories between the *Wolbachia* strains and *A. vulgare*^69,72,73^. Third, the strain *w*VulC has recently been shown to protect its host against two bacterial pathogens^74^, akin to the protective phenotypes against viruses and parasites in *Drosophila* and mosquitoes, suggesting that the presence of this strain indeed affects co-infecting bacteria. Finally, a recent in-depth characterization of the microbiota in various tissues of *A. vulgare* unveiled a highly diverse bacterial community compared to many insect species, comprising more than 200 bacterial genera even in the presence of highly abundant *Wolbachia*^34,75^. Moreover, microbiota composition differed between host populations due to an important share of environmental bacteria, resulting in a complex community of intracellular and intestinal symbionts as well as environmental passengers^34,75^. Herein, we investigate the specific impact of *Wolbachia* infection on microbiota composition in *A. vulgare* using 16S rRNA gene metabarcoding and genetic fingerprinting via Temperature Gradient Gel Electrophoresis (TGGE). Our results show that, although host origin is the major determinant, *Wolbachia* infection has a noticeable and strain-specific impact on microbiota composition within each host population, resulting in reduced abundances of many bacterial taxa, possibly due to competitive interactions.

## Results

**Figure 1:**
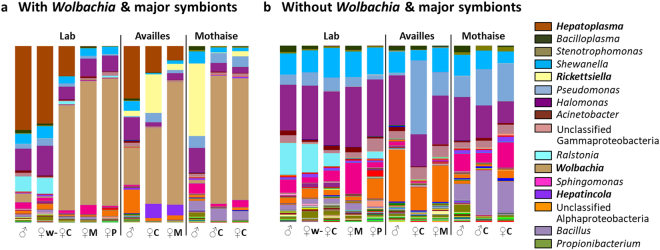
Microbiota composition (%) at the genus level depending on host origin, gender and *Wolbachia* infection. The sequencing data from five different tissues were merged in order to obtain a representative profile for each sample type. The most abundant bacterial genera are specified in the legend, their order corresponding to the order of taxa in the barplots from top to bottom. **(a)** represents the complete bacterial community including *Wolbachia* and three other highly abundant terrestrial isopod symbionts (i.e. *Hepatoplasma*, *Hepatincola*, *Rickettsiella*, highlighted in bold in the legend). These highly abundant taxa were removed in **(b)** in order to obtain a less skewed representation of the rarer taxa.

In the present study, we analysed the microbiota from a total of 77 *A. vulgare* collected from four laboratory lineages and two field sites in France (Availles and Plaine Mothaise, Table 1). Individuals from the laboratory lineages consisted of *Wolbachia*-uninfected males and females as well as females infected with either of the three feminizing *Wolbachia* strains *w*VulC, *w*VulM and *w*VulP. Specimens from the two natural populations consisted of uninfected males and *w*VulC-infected females (both sites), as well as *w*VulM-infected females (Availles only) and a single intersex male (the result of incomplete feminization) infected with *w*VulC from the Plaine Mothaise (Table 1). 16S rRNA gene amplicons were obtained from five different tissues (haemolymph, nerve cord, gonads, midgut caeca and hindgut) and biological replicates from the same tissue and sample type (origin x gender x *Wolbachia* strain) were pooled for sequencing. This resulted in 55 amplicon pools yielding 313 457 high-quality reads clustered into 1380 OTUs represented by ≥3 reads at the 97% similarity cut-off (see Supplementary Table S1 for details). These OTUs represented 19 bacterial phyla, 34 classes and 229 genera other than *Wolbachia* (see Supplementary Table S2 for a detailed taxonomy, Fig. 1). All reads identified as *Wolbachia* were excluded from the dataset for subsequent analyses and the tissue-specific data from the same sample type were merged in order to obtain a representative “whole animal” profile (Fig. 1), yielding an average of 14 800 reads clustered into 298.5 OTUs per sample type.

**Table 1:** Origin, infection status and number of specimens used for 16S rRNA gene amplicon sequencing and/or TGGE fingerprinting. Individuals were pooled for amplicon sequencing.

	Lineage/Population	Gender (*Wolbachia* strain)	Number of Specimens	
			Sequencing	TGGE
Laboratory	*Wolbachia*-free	M	10	9
		F	10	7
	C lineage	F (*w*VulC)	10	8
	M lineage	F (*w*VulM)	10	6
	P lineage	F (*w*VulP)	10	6
Field	Availles	M	6	0
		F (*w*VulC)	6	0
		F (*w*VulM)	6	0
	Plaine Mothaise	M	3	0
		M intersex (*w*VulC)	1	0
		F (*w*VulC)	5	0

### Impact of *Wolbachia* infection on taxonomic richness and diversity

**Figure 2:**
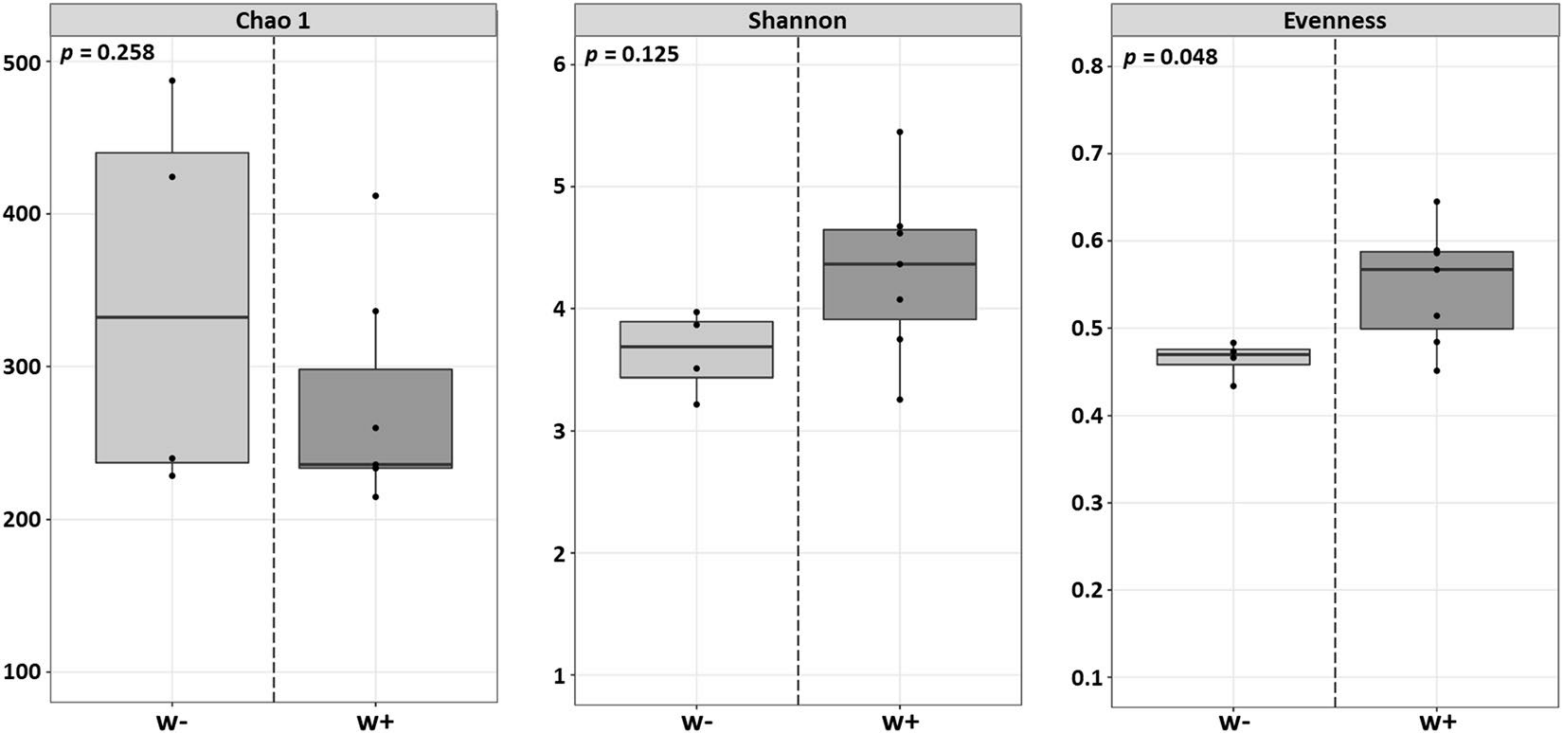
Impact of *Wolbachia* infection on bacterial taxonomic richness and diversity. Boxplots representing taxonomic richness (Chao 1), diversity (Shannon Index) and evenness. Alphadiversity indices were compared depending on *Wolbachia* infection (i.e. between *Wolbachia*-infected and uninfected isopods) using two-sample t-tests (*p*-values in the top left corner of each panel). All indices were calculated at a depth of 5000 reads after merging the sequencing data from five different tissues.

We first assessed whether *Wolbachia* infection affected bacterial taxonomic richness, diversity and community evenness, as estimated by the species richness estimator Chao1 and the Shannon Indices of diversity and evenness, respectively. Taxonomic richness and diversity were not significantly different between *Wolbachia*-free and *Wolbachia*-infected isopods (Fig. 2). However, there was a tendency towards a higher evenness in the bacterial communities from *Wolbachia*-infected animals (two sample t-test *p* = 0.048) (Fig. 2). These results suggest that *Wolbachia* infection did not affect species richness but may have induced changes in the abundance of certain bacterial taxa, resulting in more even bacterial communities in the presence of *Wolbachia*.

### Impact of *Wolbachia* infection on microbiota composition

**Figure 3:**
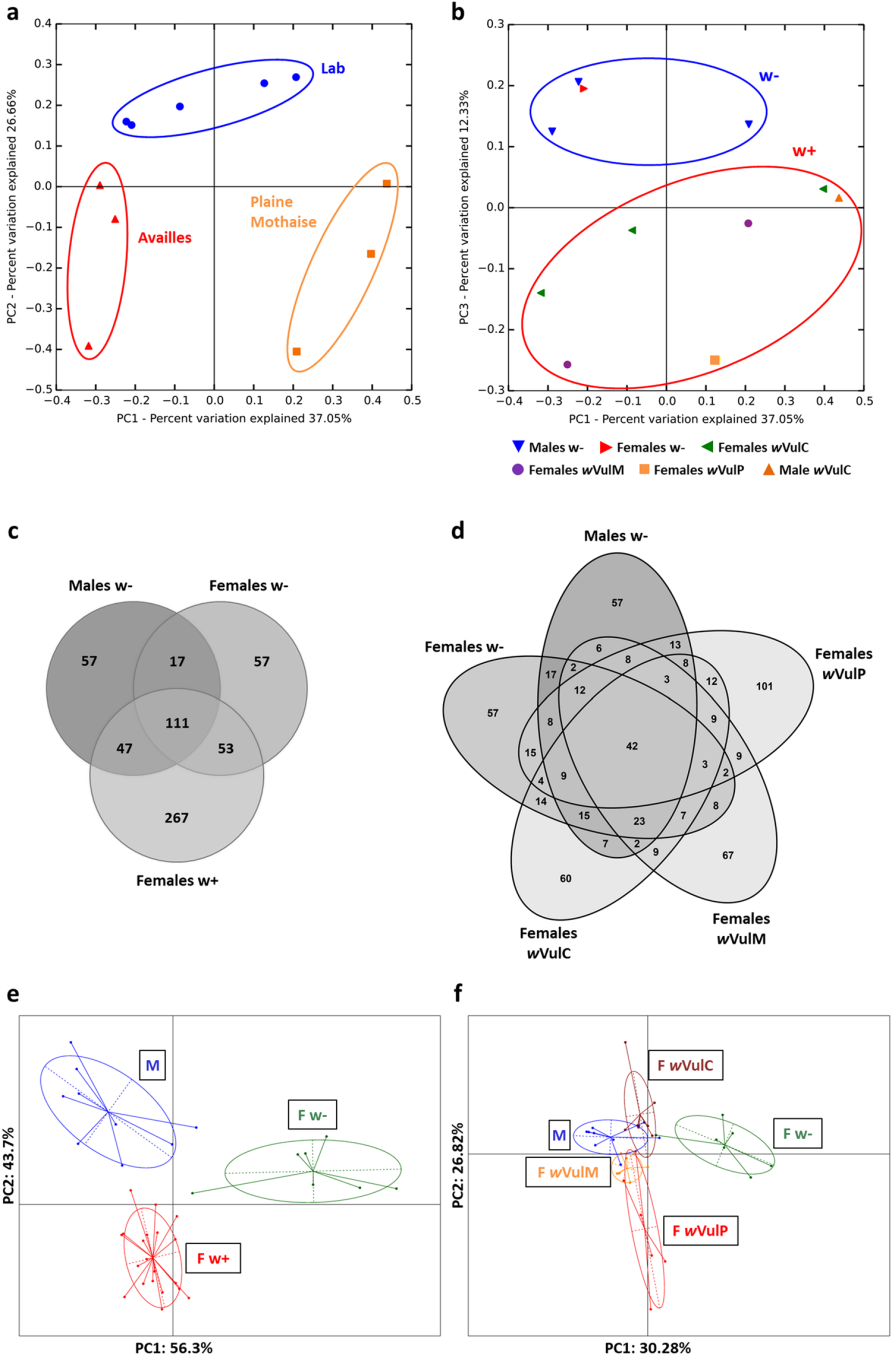
Impact of *Wolbachia* infection on microbiota composition. (**a**,**b**) PCoA based on Bray-Curtis distances showing the impact of host origin (**a**) and *Wolbachia* infection (**b**) on microbiota composition. Each data point represents the microbiota of a given sample type after merging the sequencing data from five different tissues. (**c**,**d**) Distribution of OTUs in isopods from laboratory lineages depending on *Wolbachia* infection **(c)** and infection with different *Wolbachia* strains **(d**). **(e**,**f**) Between-Class-Analysis of TGGE profiles showing differences in microbiota composition depending on *Wolbachia* infection (**e**) and infection with different *Wolbachi*a strains (**f**) for specimens from laboratory lineages. Each data point represents the merged profile from five different tissues of an individual isopod.

Taking into account that host origin is known to be a major factor shaping microbiota composition in terms of presence/absence of bacterial phylotypes in *A. vulgare*^75^, we first tested whether *Wolbachia* infection also had an impact on microbiota composition, independent of the host population (i.e. Laboratory, Availles, Plaine Mothaise). Principal Coordinates Analysis (PCoA) based on Bray-Curtis distances confirmed that host origin was indeed the major factor determining microbiota composition, with the first 2 principal components together explaining 63.71% of the variation (Fig. 3a). Nonetheless, *Wolbachia* infection was an additional factor influencing bacterial community composition, as the third principal component (explaining 12.33% of the variation) discriminated between *Wolbachia*-infected and uninfected isopods (Fig. 3b). In order to further investigate the impact of *Wolbachia* infection on microbiota composition independent of host origin, we next focused specifically on isopods from the laboratory lineages, since these (i) had been reared under controlled environmental conditions, (ii) had received the same food sources, and (iii) harboured all three feminizing *Wolbachia* strains. Interestingly, 43.84% (267/609) of the OTUs observed in these specimens occurred exclusively in *Wolbachia*-infected females, while only 9.36% (57/609) of the OTUs were specific for uninfected males and females, respectively, and 18.23% (111/609) were shared between all samples, independent of gender and infection status (Fig. 3c). Considering also the different *Wolbachia* strains, a higher number of OTUs (N = 101) were observed specifically in females infected with *w*VulP, compared with females harbouring *w*VulC (N = 60) or *w*VulM (N = 67) (Fig. 3d). These results indicate that not only *Wolbachia* infection itself, but also the different *Wolbachia* strains impact microbiota composition in *A. vulgare* under the same environmental conditions.

Considering that the sequencing data represented pooled amplicons from several individuals and therefore did not allow us to investigate inter-individual variations, we complemented the metabarcoding data with individual bacterial community profiles for the same specimens from the laboratory lineages using Temperature Gradient Gel Electrophoresis (TGGE) of the V3 region of the 16S rRNA gene. A total of 46 distinct bands (excluding the band corresponding to *Wolbachia*) were observed across all TGGE profiles, but none of these bands was found in all individuals. Indeed, the majority of bands occurred only in a relatively low number of individuals (mean ± SE = 3.52 ± 0.33 individuals) and the band corresponding to *Wolbachia* was detected at the highest frequency, being present in the 20 infected females. This suggests a relatively high level of inter-individual variation in microbiota composition in *A. vulgare*. Between-Class-Analysis of these profiles based on gender and *Wolbachia* infection resulted in three distinct clusters corresponding to the microbiotas of uninfected males, uninfected females and *Wolbachia*-infected females (Fig. 3e). When considering also the different *Wolbachia* strains, the TGGE profiles of females infected with either of the three *Wolbachia* strains formed separate clusters (Fig. 3f and Supplementary Figure S1). Interestingly, the microbiota of *w*VulM-infected females formed a tight cluster at the intersection between the two more variable clusters representing the microbiotas of females harbouring *w*VulC or *w*VulP (Fig. 3f and Supplementary Figure S1). Overall, the microbiota of *Wolbachia*-infected females appeared to be more similar to the microbiota of uninfected males than to those of uninfected females. However, Supplementary Figure S1 (an interactive 3D version of Fig. 3f) shows that uninfected males and uninfected females were discriminated by the first principal component, while *Wolbachia*-infected females and uninfected males were discriminated by the third principal component. The percentage of variation explained by the first three principal components was very similar (PC1: 30.28%, PC2: 26.82%, PC3: 23.26%), suggesting that both *Wolbachia* infection and host gender may influence microbiota composition. Unfortunately, since to date there is no reliable method to determine the genetic sex of *A. vulgare*, we could not determine which of the *Wolbachia*-infected individuals used in this study were indeed feminized genetic males.

### Identification of differentially abundant bacterial taxa

**Figure 4:**
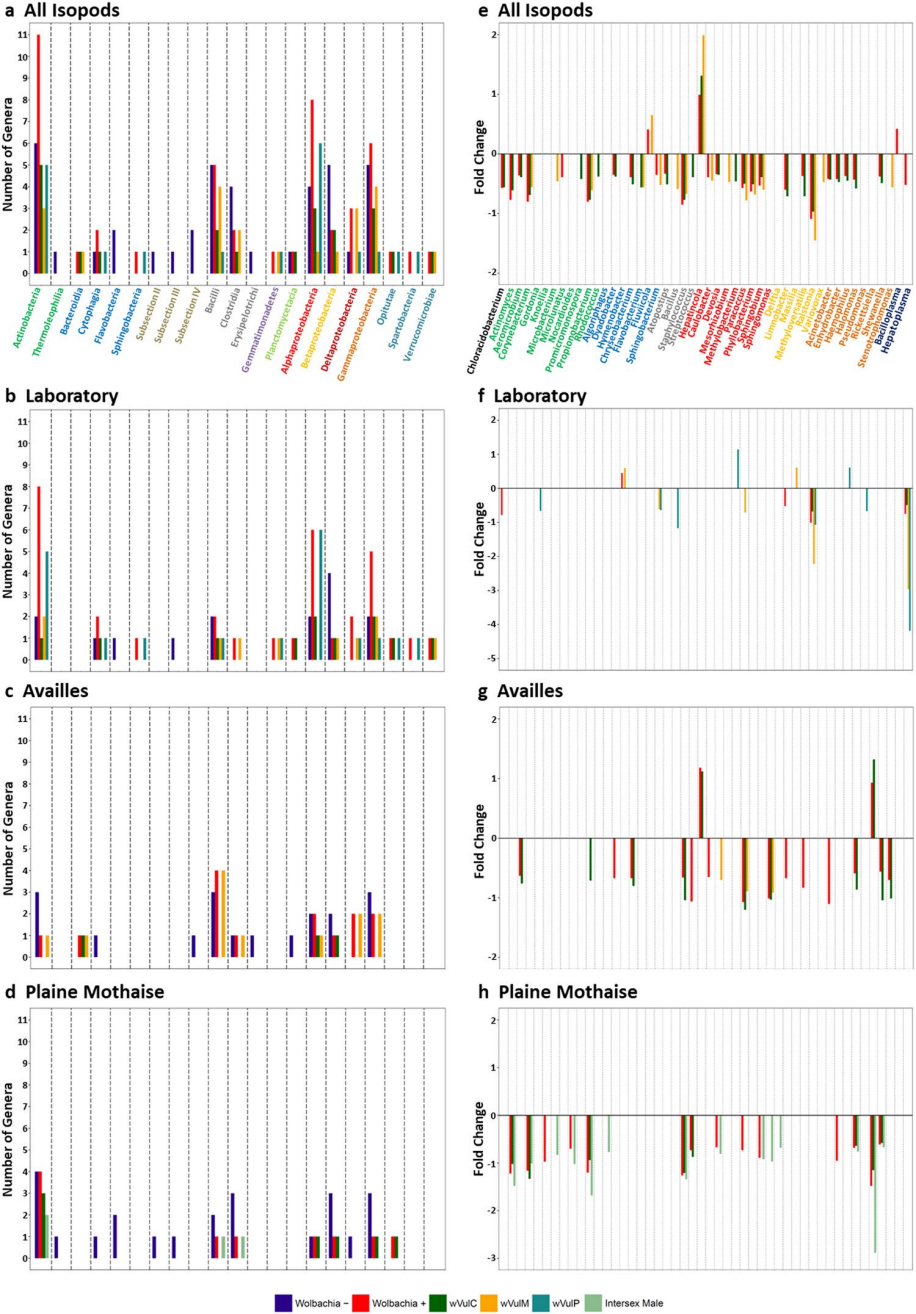
Differentially abundant bacterial taxa. (**a**–**d**) Histograms showing the distribution of genera per bacterial class which were specifically present or absent depending on *Wolbachia* infection, for all isopods independent of host origin (**a**) as well as for each population separately (**b**–**d**). (**e**–**h**) show the fold changes of the 48 genera identified as differentially abundant depending on *Wolbachia* infection using DESeq. 2, for all isopods independent of host origin (**e**) as well as for each population separately (**f**–**h**). The taxonomic identifications shown in (**a**) and (**e**) apply to all other panels and are colour-coded depending on bacterial phylum (or class for Proteobacteria): Acidobacteria = black, Actinobacteria = dark green, Bacteroidetes = light blue, Cyanobacteria = brown, Firmicutes = grey, Gemmatimonadetes  = purple, Planctomycetes = light green, Alphaproteobacteria = red, Betaproteobacteria = light orange, Deltaproteobacteria = dark red, Gammaproteobacteria = dark orange, Tenericutes = dark blue, Verrucomicrobia = Cyan.

To investigate which bacterial phylotypes within the highly diverse isopod microbiota were specifically affected by *Wolbachia* (both positively and negatively), we identified bacterial genera which were either (i) present or absent depending on *Wolbachia* infection or (ii) present in both *Wolbachia*-infected and uninfected isopods but differentially abundant based on DESeq. 2 analysis^76^. This was done for each population separately, but also after combining the data from all host populations in order to identify more general patterns (Fig. 4). For the latter, we only considered the strains *w*VulC and *w*VulM, which occurred in isopods from at least two different origins.

86 out of the 229 identified genera (37.55%) were indeed systematically present or absent depending on *Wolbachia* infection across all isopod populations (Supplementary Table S3). However, these genera were all of low abundance and together accounted for only 0.47% of all reads. Moreover, only four of these genera were observed in all host populations (*Amycolatopsis* (Actinobacteria), *Spirosoma* (Bacteroidetes), *Granulicatella* (Firmicutes) and *Cupriavidus* (Betaproteobacteria)) and all four were only present in *Wolbachia*-free isopods. Despite the fact that the other genera did not occur in all host populations, several patterns emerged when looking at higher taxonomic levels: Hence, genera belonging to the phylum Cyanobacteria and to the classes Thermoleophilia (phylum Actinobacteria), Flavobacteria (Bacteroidetes) and Erysepilotrichi (Firmicutes) were only observed in *Wolbachia*-free isopods, while genera belonging to the phyla Gemmatimonadetes and Verrucomicrobia as well as to the classes Bacteroidia and Sphingobacteria (Bacteroidetes) were only observed in *Wolbachia*-infected specimens (Fig. 4a). Moreover, a higher number of genera belonging to the Actinobacteria and Alphaproteobacteria were specifically associated with *Wolbachia*-infected isopods, while more genera of the Betaproteobacteria were only present in *Wolbachia*-free isopods (Fig. 4a). Interestingly, most of the specifically *Wolbachia*-associated genera of the Actinobacteria were observed in isopods from the laboratory lineages and the Plaine Mothaise population, i.e. primarily in specimens infected with the *w*VulC or *w*VulP strains (Fig. 4a–d). Similarly, 75% of the Alphaproteobacteria genera specifically present in *Wolbachia*-infected animals only occurred in isopods harbouring *w*VulC or *w*VulP (Fig. 4a). In contrast, more genera of the Bacilli (Firmicutes) and Deltaproteobacteria specifically present in *Wolbachia*-infected animals occurred in isopods infected with *w*VulM from the Availles population (Fig. 4a,c).

In addition, 48 genera (20.96%) were found to be differentially abundant depending on *Wolbachia* infection across all populations, mostly belonging to the Actinobacteria (11 genera), Alphaproteobacteria (9 genera) and Gammaproteobacteria (8 genera) (Fig. 4a, Supplementary Table S4). In contrast to the genera found to be specifically present or absent, the differentially abundant genera accounted for 87.54% of all reads, due to several highly abundant genera (i.e. *Propionibacterium* (Actinobacteria), *Bacillus* (Firmicutes), *Ca*. Hepatincola and *Sphingomonas* (Alphaproteobacteria), *Ralstonia* (Betaproteobacteria), *Halomonas*, *Pseudomonas*, *Rickettsiella* and *Shewanella* (Gammaproteobacteria) and *Ca*. Hepatoplasma (Tenericutes)). Both *Wolbachia* infection as well as different *Wolbachia* strains shaped bacterial abundance patterns, since more differentially abundant genera were detected in *w*VulC-infected isopods than in those harbouring *w*VulM (*w*VulC: 27, *w*VulM: 17). This was due to a higher number of differentially abundant genera belonging to the Actinobacteria and Gammaproteobacteria in *w*VulC-infected animals from the Plaine Mothaise and Availles, respectively (Fig. 4c,d). However, differences due to host origin were also apparent. Thus, more differentially abundant genera were detected in isopods from the two field populations than in specimens from laboratory lineages (Laboratory: 13, Availles: 18, Plaine Mothaise: 20) (Fig. 4b–d).

Several taxa need to be highlighted since they are well-known isopod symbionts: *Ca*. Hepatoplasma crinochetorum (Mollicutes) and *Ca*. Hepatincola porcellionum (Alphaproteobacteria), both facultative extracellular symbionts of the midgut caeca^34,77–79^, and the bacterial pathogen *Rickettsiella* (Gammaproteobacteria)^34,80–82^. Each of these genera was identified as differentially abundant depending on *Wolbachia* infection in certain host populations: *Ca*. Hepatoplasma was found to decrease in abundance in *Wolbachia*-infected laboratory lineages (particularly in the lineages harbouring *w*VulM and *w*VulP), *Ca*. Hepatincola and *Rickettsiella* increased in abundance in *w*VulC-infected isopods from Availles and *Rickettsiella* decreased in abundance in *Wolbachia*-infected specimens from the Plaine Mothaise (Fig. 4b–d). However, at least in the case of *Ca*. Hepatoplasma and *Rickettsiella*, it is more likely that this result is due to a sampling artefact rather than an actual interaction with *Wolbachia*. Indeed, *Ca*. Hepatoplasma is known to occur in the laboratory lineages derived from the *A. vulgare* population initially sampled in Helsingör (Denmark), while being almost absent from the two lineages derived from French populations^71,75^, which explains the observed statistically significant decrease in abundance. Moreover, a previous study found no differences in *Hepatoplasma* titer based on quantitative PCR between the *Wolbachia*-free and *w*VulC-infected laboratory lineages (i.e. all lineages established from the same Danish population)^75^. Similarly, only a single male from the Plaine Mothaise population was infected with *Rickettsiella* while all females were found uninfected based on diagnostic PCR, which again explains the statistically significant decrease in abundance in *Wolbachia*-infected isopods from this population but not the increase in abundance in the Availles population, where *Rickettsiella* infection is more widespread in both males and females^75^. Hence, it remains to be investigated whether *Hepatincola* and *Rickettsiella* do indeed interact with *Wolbachia*.

## Discussion

The terrestrial isopod *Armadillidium vulgare* harbours a highly diverse bacterial community consisting of ≥200 bacterial genera^34,75^ and is frequently infected by feminizing *Wolbachia*^26,69,83^. Apart from the reproductive manipulation, these *Wolbachia* have profound effects on host life history traits, e.g. reducing fecundity and immunocompetence^72,73^. In addition, the feminizing strain *w*VulC has recently been shown to protect its host against two bacterial pathogens^74^, indicating that *Wolbachia* can affect other co-infecting bacteria in this species. Using *A. vulgare* and its three feminizing *Wolbachia* strains (*w*VulC, *w*VulM, *w*VulP) as a model system, the present study shows for the first time that *Wolbachia* not only interacts with certain bacterial pathogens, viruses or eukaryotic parasites, but can even influence a diverse bacterial community as a whole under both laboratory and natural conditions. While *Wolbachia* infection had no impact on bacterial taxonomic richness and diversity, it influenced the taxonomic composition of the microbiota and, more importantly, the abundance of many bacterial taxa. Thus, 48 genera (20.96% of all identified genera), mainly belonging to the phyla Actinobacteria and Proteobacteria, were found to be differentially abundant depending on *Wolbachia* infection. Moreover, different *Wolbachia* strains also had different impacts on microbiota composition. In particular, a higher number of differentially abundant genera were detected in *w*VulC-infected isopods than in those harbouring *w*VulM, especially in specimens from a natural population in which both strains were present. This is of interest in light of previous studies suggesting that these two *Wolbachia* strains represent different co-evolutionary histories with *A. vulgare* and that *w*VulC has higher virulence, transmission rate and feminizing capacity than *w*VulM^69,84^. *w*VulM also reaches lower titers than *w*VulC in most host tissues^71^, which may explain both its lower virulence and the weaker impact on other bacterial taxa observed here. The *w*VulP strain is presumably the result of a recombination event between *w*VulC and *w*VulM, with *w*VulC as its major parent^67^. While *w*VulP has indeed similar tissue-specific titers as *w*VulC^71^, the microbiota of the laboratory-reared isopods harbouring this strain was clearly different from lineages infected with *w*VulC or *w*VulM, due to several genera belonging to the Actinobacteria and Alphaproteobacteria which were specifically associated with the *w*VulP lineage. Unfortunately, we did not find any isopods harbouring this strain in natural populations to corroborate this effect in a different host genetic background.

Nonetheless, it has to be pointed out that all the genera which were specifically present or absent and most genera identified as differentially abundant depending on *Wolbachia* infection were low-abundance genera, making it less likely that the observed *Wolbachia*-mediated changes could be sufficiently strong to influence the performance of terrestrial isopods as keystone species in soil ecosystems. Interestingly, several better-studied and highly abundant terrestrial isopod facultative symbionts (*Ca*. Hepatoplasma crinochetorum (Mollicutes), *Ca*. Hepatincola porcellionum (Alphaproteobacteria) and *Rickettsiella* (Gammaproteobacteria)) were also identified as differentially abundant depending on *Wolbachia* infection. While this is likely a sampling artefact in the case of *Ca*. Hepatoplasma, potentially a nutritional symbiont enhancing host survival on low-quality diets^85^, it remains to be investigated using specific quantitative methods whether *Ca*. Hepatincola and *Rickettsiella* (a deadly isopod pathogen) are indeed more abundant in *Wolbachia*-infected individuals. If this were the case, it could suggest some kind of facilitation due to the presence of *Wolbachia* (e.g. reduction in host immunocompetence^72,73^) and the absence of *Wolbachia*-mediated resistance against the natural isopod pathogen *Rickettsiella*. Alternatively, *Rickettsiella* might reach higher titers in *Wolbachia*-infected individuals due to a *Wolbachia*-mediated tolerance as previously observed in *D. simulans*^60^, allowing the host to survive with higher pathogen loads compared to *Wolbachia*-free specimens.

Since the first discoveries of *Wolbachia*-mediated protection against pathogens in *Drosophila* and mosquitoes, it has been hypothesized that these protective phenotypes might be due to (i) an enhanced immune response, especially in transfected non-native hosts^57,86,87^, or (ii) competition between *Wolbachia* and other microorganisms for resources and space in the shared host environment, resulting in titer-dependent protection^60,88–90^. Several factors make it more likely that the observed differences in the microbiotas of *Wolbachia*-infected *A. vulgare* are predominantly due to competitive interactions: (i) Previous studies demonstrated a reduction in several immune effectors in *Wolbachia*-infected isopods, arguing against immune priming^72,73^, (ii) total bacterial loads increase in some, but not all tissues of *Wolbachia*-infected individuals, suggesting a competition for resources or space between *Wolbachia* and other bacteria^71^, and (iii) in line with the latter, the present study shows that most differentially abundant bacterial phylotypes indeed decreased in abundance.

Based on the data presented here and in our previous study^75^, *Wolbachia* infection is not the only factor shaping microbiota composition in *A. vulgare*. In particular, host origin had been previously identified as a major factor determining the taxonomic composition of the microbiota in this species^75^. While this could be due to both host genotype and/or different environments (e.g. in terms of soil or food-associated bacteria), we argue that the latter is the more important driver in *A. vulgare*. First, although the laboratory lineages used in this study were established from three different natural populations with very different genotypes^71^, their microbiotas are more homogenous in taxonomic composition after many years of controlled laboratory rearing on the same food sources than their conspecifics from natural populations^75^. This pattern was also obvious in the present study, since different bacterial presence/absence and differential abundance patterns were observed for each population. Second, environmental bacteria have an important share in the taxonomic richness of the microbiota in *A. vulgare*, potentially acquired from the prevailing food sources or the surrounding soil environment^75^. This has been corroborated by a recent study in the terrestrial isopod *Porcellio scaber*, whose microbiota composition was found to vary depending on different plant food sources in a controlled feeding experiment^91^. Host gender may be an additional confounding factor, especially in the present case where (i) only females harbour *Wolbachia*, (ii) it is impossible to distinguish *Wolbachia*-infected genetic females from feminized genetic males since no sex-specific markers are currently available for terrestrial isopods, and (iii) uninfected genetic females are rare in infected populations. Hence, it is impossible to determine which of the *Wolbachia*-infected specimens used in this study were genetically male or female, thus precluding any firm conclusions regarding the genetic sex as a driver of microbiota composition. Nonetheless, our data indicate that *Wolbachia* infection has a distinct impact on microbiota composition, independent of the genetic sex of the host: While the microbiota of uninfected females from the laboratory could not always be distinguished from that of uninfected males of the same genotype (i.e. the two microbiotas had distinct TGGE profiles but clustered together in PCoA of 16S rRNA gene amplicon data, which is a much more powerful technique to capture bacterial diversity), the microbiota associated with *w*VulC-infected females from the same initial population was different from both uninfected males and females in both analyses. Moreover, the microbiotas of uninfected males from several populations were different from those of infected females (which may be feminized genetic males), indicating a more general trend independent of host genotype, host genetic sex or environmental factors. Lastly, the microbiota of the *Wolbachia*-infected intersex male was most similar to the microbiota of infected females from the same natural population instead of clustering with the uninfected males.

Taken together, the composition of the bacterial microbiota associated with *A. vulgare* appears to be shaped by several factors: Host origin (via environmental bacteria and nutrition), *Wolbachia* infection status and host gender. While the respective impacts of these factors are not easily disentangled, similarly complex multifactorial patterns likely underlie many animal-bacteria symbioses under ecologically realistic conditions.

## Methods

### Terrestrial isopod samples and *Wolbachia* infection status

The 77 *Armadillidium vulgare* used in this study were sampled from four laboratory lineages and two field sites in France (Table 1) and were partly the same as those used in previous studies^71,75^. In the laboratory, animals were reared at 20 °C and natural photoperiod in plastic breeding boxes on moistened potting mix and fed *ad libitum* with lime tree leaves and carrot slices. One laboratory lineage was *Wolbachia*-free, i.e. both males and females from this lineage do not carry *Wolbachia*. In the other three lineages, natural infections with either of the *Wolbachia* strains *w*VulC, *w*VulM or *w*VulP have been stably maintained for at least 7 years (30 years for the oldest lineage). The *Wolbachia*-free lineage and the *w*VulC lineage were established from the same original population sampled in Helsingör, Denmark in 1982. The other two lineages carrying *w*VulM or *w*VulP derive from specimens sampled in Mery-sur-Cher (France) in 1999 and in Poitiers (France) in 2007, respectively. 10 males and 10 females (pairs of brothers and sisters) were randomly chosen from the *Wolbachia*-free lineage, as well as 10 females each from the *w*VulC, *w*VulM and *w*VulP lineages. Additional isopods from two natural populations in France (Availles-Thouarsais (46° 51′ 37′′N, 0° 8′ 28′′E) and Plaine Mothaise (46° 21′ 21′′N, 0° 06′ 32′′E)) were sampled in autumn 2011 and 2012. The collected specimens were kept in plastic boxes with soil and leaves from their respective sampling site until dissection.

Prior to dissection, all isopods were surface-sterilized using sodium hypochlorite and haemolymph was collected after piercing the dorsal cuticle with a sterile needle. For each specimen, DNA was extracted from the collected haemolymph and four different tissues (gonads, nerve cord, midgut caeca and hindgut) using phenol–chloroform^92^. *Wolbachia* infection status as well as *Wolbachia* titers in all host tissues of the specimens used in this study have been determined previously using diagnostic as well as quantitative PCR of the *wsp* gene^71,75^. Females collected from Availles harboured either *w*VulC or *w*VulM, while all females collected from the Plaine Mothaise were infected with *w*VulC. In addition, one individual from the Plaine Mothaise was identified as an intersex male infected with *w*VulC: While the external morphological characters were male, the androgenic glands were hypertrophied, which is an indication of *Wolbachia* infection with incomplete feminization^93,94^.

### 16S rRNA gene metabarcoding

The 16S rRNA gene amplicon sequences analysed in this study derive from the same pyrosequencing dataset used for the initial characterization of the microbiota of *A. vulgare* in different host tissues and populations^75^ (Accession Number PRJEB8160). Briefly, a 526 bp-fragment spanning the variable regions V1-V3 of the 16S rRNA gene was amplified using the universal primers 27 F and 520 R. Primers were adapted for 454 pyrosequencing by adding the 454 Adapter A and a 10-bp Multiplex Identifier sequence (MID) to the reverse primer 520 R as well as the 454 Adapter B to the forward primer 27 F. Amplicons were obtained from five different tissues (haemolymph, nerve cord, gonads, midgut caeca and hindgut) and up to 10 biological replicates from the same tissue and sample type (origin x gender x *Wolbachia* strain) were pooled for sequencing, resulting in 55 amplicon pools (see Supplementary Table S1 for details). The amplicon pools were purified using AMPure Beads (Agencourt Bioscience Corporation), quantified using PicoGreen (Invitrogen) and sequenced on a 454 GS FLX sequencer (Roche, 454 Life Sciences) by GenoScreen (Lille, France) as well as on a GS Junior sequencer (Roche, 454 Life Sciences) at the University of Poitiers (France).

### Metabarcoding Data Analysis

The 16S rRNA gene pyrotags were analysed using QIIME version 1.9.1^95^ and R (R Project 3.3.2). Briefly, the flowgrams were denoised with AmpliconNoise^96,97^ and chimeras were removed using Perseus^97^. All reads shorter than 250 bp were discarded and the remaining reads were clustered into Operational Taxonomic Units (OTUs) at 97% similarity using uclust^98^. Representative sequences were aligned against the Silva reference alignment (release 108,^99^) using PyNAST^100^ and identified using the RDP Classifier^101^. Rare OTUs (i.e. singletons and doubletons) were discarded, resulting in 1380 OTUs represented by ≥3 reads (see Supplementary Table S1 for details). All reads identified as *Wolbachia* were excluded from the dataset for subsequent analyses and the data from the five different tissues of the same sample type were merged in order to obtain a representative “whole animal” profile. Taxonomic richness, diversity and evenness were determined using the nonparametric species richness estimator Chao 1 and the Shannon Index of diversity and evenness, after subsampling of 5000 sequences per sample. Alpha diversity indices were compared between *Wolbachia*-infected and uninfected isopods using two-sample t-tests with 1000 Monte Carlo permutations. Betadiversity was analysed using Principal Coordinates Analysis (PCoA) based on Bray-Curtis distances. Venn diagrams were produced in R using the VennDiagram package. Differentially abundant taxa were determined after data normalization using the DESeq. 2 Wald Test^76^ as implemented in QIIME.

### Temperature Gradient Gel Electrophoresis (TGGE)

Considering that the sequencing data from pooled amplicons did not allow us to investigate inter-individual variations, the metabarcoding data was complemented with individual bacterial community profiles from the same specimens from the laboratory lineages using Temperature Gradient Gel Electrophoresis (TGGE). A 196 bp-fragment of the variable region V3, also included in the fragment used for amplicon sequencing, was amplified from the tissue samples using a nested PCR approach: First, a 795-bp fragment was amplified using primers 27 F and 786 R^102^, followed by a second amplification targeting the V3 region using primers 338F-GC and 520 R^103^. Primer 338 F contained a 42-nucleotide GC-clamp preventing the complete denaturation of the DNA strands during TGGE^103^. All PCR amplifications were confirmed by electrophoresis in 1.5% agarose gels. TGGE was performed using the DCode Universal Mutation Detection System™ (Bio-Rad) following a protocol modified from^104^. Briefly, 20 μl of the final PCR products were run across a temperature gradient from 38 °C to 70 °C (ramping of 1.3 °C/h) at 60 V on 10% polyacrylamide gels. A standard containing V3 16S rRNA gene fragments from several reference bacteria (*Bacillus megaterium*, *Escherichia coli*, *Listeria ivanovii*, *Micrococcus luteus*, *Salmonella typhimurium* and *Wolbachia* spp.) was loaded on each gel to allow the standardization of bands between gels. This also allowed a visual screening of *Wolbachia* infection across all samples via the presence of a band at the position corresponding to *Wolbachia* spp. in the standard. After electrophoresis, gels were stained with ethidium bromide and photographed under UV. Banding patterns on each gel were standardized based on the position of the reference bands using the GelAnalyzer 2010 software (www.gelanalyzer.com). Banding patterns across all gels were then analysed using an in-house Perl script: Bands with highly similar positions across all gels were grouped into a single normalized band, resulting in a presence/absence matrix of each normalized band per tissue sample. Finally, bacterial community profiles of each individual were established by merging the tissue-specific profiles into a single presence/absence profile per individual. The band corresponding to *Wolbachia* was removed from the dataset and bacterial community composition was analysed via principal component analysis (PCA) followed by between-class analysis (BCA)^105^ in R using the ade4 package. The 3D image of the BCA was made using the function scatter3d of the car package.

### Data availability

The 16S rRNA gene dataset used in this study is accessible in the European Nucleotide Archive under the Accession Number PRJEB8160.

## Electronic supplementary material

Table S1, S3 & S4

Table S2

Supplementary information Figure S1
