# Supplementary material for: Feminizing Wolbachia influence microbiota composition in the terrestrial isopod Armadillidium vulgare
Source: Sci Rep. 2018 May 3;8:6998. doi: 10.1038/s41598-018-25450-4 (PMC5934373; doi:10.1038/s41598-018-25450-4)
Supplement: Supplementary file 1 — Table S1, S3 & S4 [file 41598_2018_25450_MOESM1_ESM.zip › Supplementary Material.pdf]

Feminizing *Wolbachia* influence microbiota composition in the terrestrial isopod *Armadillidium vulgare*

Jessica Dittmer[1,2] & Didier Bouchon[1]

[1]Université de Poitiers, UMR CNRS 7267, Ecologie et Biologie des Interactions, équipe Ecologie Evolution Symbiose, 5 rue Albert Turpin, 86073 Poitiers, France

[2]Current address: Dipartimento di Biologia e Biotecnologie, Università degli Studi di Pavia, Via Ferrata 9, 27100 Pavia, Italy

Supplementary Material

Supplementary Table S1 Summary of the amplicon pools, including sequence yield and estimates of bacterial richness and diversity. Whole animal data (highlighted in bold) were obtained by merging the reads from the different tissues and do not represent actual whole body samples.

Origin	Condition		No of individuals	No of Reads[a]	No of OTUs (observed richness)[a]	Richness and diversity estimates[b]	
	Gender + Infection Status	Tissue				Chao 1	Shannon
Lab w- lineage	Male w-	Haemolymph	10	3714	112	118.93	3.35
Lab w- lineage	Male w-	Gonads	10	2994	105	112.98	3.29
Lab w- lineage	Male w-	Nerve Cord	10	3749	93	103.33	3.11
Lab w- lineage	Male w-	Caeca	10	2807	20	26.83	0.64
Lab w- lineage	Male w-	Gut	10	4684	135	146.15	3.43
**Lab w- lineage**	**Male w-**	**Whole animal**	**10**	**17948**	**234**	**209.79**	**3.31**
Lab w- lineage	Female w-	Haemolymph	10	3104	115	127.20	3.85
Lab w- lineage	Female w-	Gonads	10	3885	132	138.93	4.16
Lab w- lineage	Female w-	Nerve Cord	10	3128	108	126.68	3.59
Lab w- lineage	Female w-	Caeca	10	3338	29	32.72	0.80
Lab w- lineage	Female w-	Gut	10	2814	99	109.42	3.00
**Lab w- lineage**	**Female w-**	**Whole animal**	**10**	**16269**	**239**	**215.18**	**3.49**
Lab C lineage	Female *w*VulC	Haemolymph	10	3792	137	146.86	3.83
Lab C lineage	Female *w*VulC	Gonads	10	4449	36	46.25	0.63
Lab C lineage	Female *w*VulC	Nerve Cord	10	4730	86	96.48	1.82
Lab C lineage	Female *w*VulC	Caeca	10	3518	75	80.33	2.62
Lab C lineage	Female *w*VulC	Gut	10	4339	99	103.98	2.06
**Lab C lineage**	**Female *w*VulC**	**Whole animal**	**10**	**20828**	**234**	**215.73**	**2.45**
Lab M lineage	Female *w*VulM	Haemolymph	10	3118	95	106.74	2.61
Lab M lineage	Female *w*VulM	Gonads	10	5119	63	75.08	0.85
Lab M lineage	Female *w*VulM	Nerve Cord	10	4697	106	121.00	2.34
Lab M lineage	Female *w*VulM	Caeca	10	4314	106	113.90	2.98
Lab M lineage	Female *w*VulM	Gut	10	5457	93	102.75	1.33
**Lab M lineage**	**Female *w*VulM**	**Whole animal**	**10**	**22705**	**226**	**208.15**	**2.13**

Supplementary Table S1 (cont.) Summary of the amplicon pools, including sequence yield and estimates of bacterial richness and diversity. Whole animal data (highlighted in bold) were obtained by merging the reads from the different tissues and do not represent actual whole body samples.

Origin	Condition		No of individuals	No of Reads[a]	No of OTUs (observed richness)[a]	Richness and diversity estimates[b]	
	Gender + Infection Status	Tissue				Chao 1	Shannon
Lab P lineage	Female *w*VulP	Haemolymph	10	4237	142	152.96	2.67
Lab P lineage	Female *w*VulP	Gonads	10	5136	77	82.89	1.23
Lab P lineage	Female *w*VulP	Nerve Cord	10	4598	104	114.32	1.76
Lab P lineage	Female *w*VulP	Caeca	10	4725	88	97.36	2.16
Lab P lineage	Female *w*VulP	Gut	10	4176	92	103.64	2.13
**Lab P lineage**	**Female *w*VulP**	**Whole animal**	**10**	**22872**	**268**	**220.69**	**2.11**
Availles	Male w-	Haemolymph	6	4652	268	302.63	4.74
Availles	Male w-	Gonads	6	6408	142	140.30	2.83
Availles	Male w-	Nerve Cord	6	5484	176	197.66	3.21
Availles	Male w-	Caeca	6	5268	84	104.09	2.02
Availles	Male w-	Gut	6	5624	210	230.34	3.72
**Availles**	**Male w-**	**Whole animal**	**6**	**27436**	**443**	**385.71**	**3.76**
Availles	Female *w*VulC	Haemolymph	6	5892	166	185.10	3.02
Availles	Female *w*VulC	Gonads	6	11116	85	92.96	1.18
Availles	Female *w*VulC	Nerve Cord	6	8321	113	126.96	2.37
Availles	Female *w*VulC	Caeca	6	8031	50	53.18	1.94
Availles	Female *w*VulC	Gut	6	7601	116	115.92	2.89
**Availles**	**Female *w*VulC**	**Whole animal**	**6**	**40961**	**258**	**179.25**	**2.87**
Availles	Female *w*VulM	Haemolymph	6	6120	172	220.59	2.31
Availles	Female *w*VulM	Gonads	6	15152	102	83.75	0.51
Availles	Female *w*VulM	Nerve Cord	6	9279	180	167.91	2.09
Availles	Female *w*VulM	Caeca	6	7680	129	129.04	2.56
Availles	Female *w*VulM	Gut	6	11474	149	130.87	1.85
**Availles**	**Female *w*VulM**	**Whole animal**	**6**	**49702**	**339**	**223.62**	**2.17**

Supplementary Table S1 (cont.) Summary of the amplicon pools, including sequence yield and estimates of bacterial richness and diversity. Whole animal data (highlighted in bold) were obtained by merging the reads from the different tissues and do not represent actual whole body samples.

Origin	Condition		No of individuals	No of Reads[a]	No of OTUs (observed richness)[a]	Richness and diversity estimates[b]	
	Gender + Infection Status	Tissue				Chao 1	Shannon
Plaine Mothaise	Male w-	Haemolymph	3	5645	303	333.21	4.84
Plaine Mothaise	Male w-	Gonads	3	5224	221	244.72	2.93
Plaine Mothaise	Male w-	Nerve Cord	3	5989	219	240.53	3.16
Plaine Mothaise	Male w-	Caeca	3	4928	186	212.51	3.47
Plaine Mothaise	Male w-	Gut	3	7071	200	206.61	3.70
**Plaine Mothaise**	**Male w-**	**Whole animal**	**3**	**28857**	**508**	**403.53**	**4.00**
Plaine Mothaise	Male intersexual	Haemolymph	1	3970	148	162.40	3.68
Plaine Mothaise	Male intersexual	Gonads	1	5318	81	94.07	1.02
Plaine Mothaise	Male intersexual	Nerve Cord	1	6029	156	167.67	2.67
Plaine Mothaise	Male intersexual	Caeca	1	3694	90	104.96	2.16
Plaine Mothaise	Male intersexual	Gut	1	6196	87	93.27	1.05
**Plaine Mothaise**	**Male intersexual**	**Whole animal**	**1**	**25207**	**253**	**217.28**	**2.20**
Plaine Mothaise	Female *w*VulC	Haemolymph	5	6717	314	328.31	4.83
Plaine Mothaise	Female *w*VulC	Gonads	5	10560	143	132.86	0.94
Plaine Mothaise	Female *w*VulC	Nerve Cord	5	7145	251	285.26	3.31
Plaine Mothaise	Female *w*VulC	Caeca	5	7461	183	203.42	1.79
Plaine Mothaise	Female *w*VulC	Gut	5	8786	220	235.82	1.96
**Plaine Mothaise**	**Female wVulC**	**Whole animal**	**5**	**40669**	**443**	**319.59**	**2.68**

[a]Including reads/OTUs corresponding to *Wolbachia*
[b]Based on random subsampling of 2500 sequences

Supplementary Table S2 Detailed taxonomy of the bacterial phylotypes and their distribution (read counts) depending on host population, gender and *Wolbachia* infection status. The column "All populations" contains merged data from all populations.

See separate file "Supplementary Table S2_Taxonomy_Counts.xlsx"

Supplementary Table S3. Distribution of genera specifically present or absent depending on *Wolbachia* infection and different *Wolbachia* strains, for each population separately as well as independent of host origin (“All Populations”). See Supplementary Table S2 for a detailed taxonomy.

		All Populations				Laboratory					Availles				Plaine Mothaise			
Phylum	Class	*Wolbachia -*	*Wolbachia +*	*w*VulC	*w*VulM	*Wolbachia -*	*Wolbachia +*	*w*VulC	*w*VulM	*w*VulP	*Wolbachia -*	*Wolbachia +*	*w*VulC	*w*VulM	*Wolbachia -*	*Wolbachia +*	♀ *w*VulC	♂ *w*VulC
Actinobacteria	Actinobacteria	6	11	5	3	2	8	1	2	5	3	1	0	1	4	4	3	2
	Thermoleophilia	1	0	0	0	0	0	0	0	0	0	0	0	0	1	0	0	0
Bacteroidetes	Bacteroidia	0	1	1	1	0	0	0	0	0	0	1	1	1	0	0	0	0
	Cytophagia	1	2	1	0	1	2	1	0	1	1	0	0	0	1	0	0	0
	Flavobacteria	2	0	0	0	1	0	0	0	0	0	0	0	0	2	0	0	0
	Sphingobacteria	0	1	0	0	0	1	0	0	1	0	0	0	0	0	0	0	0
Cyanobacteria	Subsection II	1	0	0	0	0	0	0	0	0	0	0	0	0	1	0	0	0
	Subsection III	1	0	0	0	1	0	0	0	0	0	0	0	0	1	0	0	0
	Subsection IV	2	0	0	0	0	0	0	0	0	1	0	0	0	0	0	0	0
Firmicutes	Bacilli	5	5	2	4	2	2	1	1	1	3	4	0	4	2	1	0	1
	Clostridia	4	2	1	2	0	1	0	1	0	1	1	0	1	3	1	0	1
	Erysipelotrichi	1	0	0	0	0	0	0	0	0	1	0	0	0	0	0	0	0
Gemmatimonadetes	Gemmatimonadetes	0	1	0	1	0	1	0	1	1	0	0	0	0	0	0	0	0
Planctomycetes	Planctomycetacia	1	1	1	0	0	1	1	0	0	1	0	0	0	0	0	0	0
Proteobacteria	Alphaproteobacteria	4	8	3	1	2	6	2	0	6	2	2	1	1	1	1	1	0
	Betaproteobacteria	5	2	2	1	4	1	1	1	0	2	1	1	0	3	1	1	0
	Deltaproteobacteria	1	3	0	3	0	2	0	1	1	0	2	0	2	1	0	0	0
	Gammaproteobacteria	5	6	3	4	2	5	2	2	1	3	2	0	2	3	1	1	0
Verrucomicrobia	Opitutae	0	1	1	0	0	1	1	0	1	0	0	0	0	0	1	1	0
	Spartobacteria	0	1	0	0	0	1	0	0	1	0	0	0	0	0	0	0	0
	Verrucomicrobiae	0	1	1	1	0	1	1	1	0	0	0	0	0	0	0	0	0
	Total	40	46	21	21	15	33	11	10	19	18	14	3	12	23	10	7	4

Supplementary Table S4. Fold changes for differentially abundant bacterial taxa depending on *Wolbachia* infection (*W*), *Wolbachia* strains (*w*VulC, *w*VulM, *w*VulP) and host origin (Laboratory, Availles, Plaine Mothaise). Differential abundance was determined using DESeq2 [76]. Fields highlighted in blue indicate decreased abundance, fields highlighted in red increased abundance.

Phylum	Class	Genus	All populations			Laboratory				Availles			Plaine Mothaise		
			W	*w*VulC	*w*VulM	*W*	*w*VulC	*w*VulM	*w*VulP	*W*	*w*VulC	*w*VulM	*W*	♀ *w*VulC	♂ *w*VulC
Acidobacteria			-0.42	-0.50		-0.62									
	Acidobacteria		-0.52	-0.63		-0.76							-0.63		
		Ca. Chloracidobacterium	-0.57	-0.56		-0.78									
		Unidentified											-0.63		
Actinobacteria			-0.55	-0.52	-0.50								-1.07	-0.89	-1.32
	Actinobacteria		-0.64	-0.61	-0.61								-1.17	-0.88	-1.47
		Actinomyces	-0.77	-0.61									-1.22	-1.02	-1.48
		Aeromicrobium	-0.36	-0.39						-0.63	-0.76				
		Corynebacterium	-0.80	-0.69	-0.56								-1.16	-1.33	-1.01
		Gordonia							-0.66						
		Knoellia											-0.97		
		Microbacterium			-0.46										-0.83
		Microlunatus	-0.39												
		Nocardioides											-0.70		-1.02
		Promicromonospora		-0.42											
		Propionibacterium	-0.80	-0.77	-0.61						-0.71		-1.20	-0.94	-1.68
		Rhodococcus		-0.38											
		Unidentified				0.44			0.74						-1.03
Bacteroidetes				-0.40							-0.74				
	Cytophagia											0.68			
		Algoriphagus													-0.77
		Dyadobacter	-0.35	-0.38						-0.67					
		Hymenobacter				0.45		0.60							
		Unidentified						0.59				1.10			
	Flavobacteria		-0.41	-0.57						-0.57	-0.90				
		Chryseobacterium	-0.39	-0.51						-0.67	-0.80				
		Flavobacterium		-0.56	-0.56										
		Fluviicola	0.41		0.65										
		Unidentified	-0.44	-0.53		-0.48			-0.96		-0.88				
	Sphingobacteria										-0.83				-0.87
		Sphingobacterium	-0.35		-0.52			-0.61	-0.64						
		Unidentified									-0.70				
Candidate Division TM7													-0.96		-1.29
	Unidentified										-0.80		-1.11	-0.74	-1.51
Cyanobacteria				-0.54											
Firmicutes										-0.89	-1.90				
	Bacilli				-0.59					-0.96	-1.66				
		Atopostipes	-0.33	-0.51											
		Bacillus			-0.59				-1.17						
		Staphylococcus	-0.85	-0.77	-0.67					-0.66	-1.04		-1.26	-1.21	-1.34
		Streptococcus		-0.39						-1.06			-0.73	-0.87	
		Unidentified	0.42			0.88									
	Clostridia													-0.74	
Fusobacteria													-0.72		
	Fusobacteria												-0.79		
Planctomycetes										-0.56					
	Planctomycetacia		-0.34							-0.61					
Proteobacteria											0.50		-0.73	-0.56	-0.99
	Alphaproteobacteria														-0.96
		Ca. Hepatincola	0.99	1.31	1.99					1.18	1.12				
		Caulobacter	-0.39		-0.45					-0.65					
		Devosia	-0.34	-0.35								-0.70	-0.67		-0.81
		Mesorhizobium			-0.47										
		Methylobacterium		-0.46					1.14						
		Paracoccus	-0.57	-0.50	-0.78			-0.71		-1.07	-1.20	-0.89	-0.73		
		Phyllobacterium	-0.63	-0.51	-0.68										
		Sphingobium	-0.53	-0.39	-0.60								-0.89		-0.92
		Sphingomonas								-1.01	-1.03	-0.91			-0.97
		Unidentified	-0.48	-0.67						-0.75	-0.98				-1.00

Betaproteobacteria	-0.85	-0.72	-1.10	-1.07	-0.62	-1.49	-1.11						-0.62
Delftia													-0.68
Limnobacter	-0.60	-0.71		-0.52				-0.67					
Massilia						0.61							
Methyloversatilis	-0.37	-0.71						-0.83					
Ralstonia	-1.09	-0.97	-1.45	-1.01	-0.68	-2.22	-1.07						
Variovorax			-0.47										
Unidentified			-0.60	-0.43			-0.59						-0.65
Deltaproteobacteria		-0.46											
Gammaproteobacteria	-0.29		-0.36								-0.91	-0.74	-1.10
Acinetobacter	-0.42	-0.43						-1.10					
Enhydrobacter	-0.42	-0.47									-0.95		
Haemophilus	-0.37	-0.45					0.61						
Halomonas	-0.43	-0.58						-0.59	-0.86		-0.68	-0.64	-0.76
Pseudomonas							-0.67						
Rickettsiella								0.93	1.32		-1.48	-1.15	-2.88
Shewanella	-0.38	-0.49						-0.56	-1.04		-0.61	-0.58	-0.67
Stenotrophomonas			-0.56					-0.70	-1.01				
Unidentified					0.48								
Tenericutes	-0.50			-0.80	-0.41	-1.95	-4.63						
Mollicutes	-0.58			-1.05	-0.50	-2.41	-4.68						
Ca. Bacilloplasma	0.42												
Ca. Hepatoplasma	-0.52			-0.75	-0.49	-2.96	-4.18						

Supplementary Figure S1. Interactive 3D image of the Between-Class-Analysis of TGGE profiles showing differences in microbiota composition depending on *Wolbachia* infection status in specimens from laboratory lineages. Each data point represents the merged profile from five different tissues of an individual isopod.

See separate file "Supplementary_Figure_S1.html"
