# Supplementary material for: Feminizing Wolbachia influence microbiota composition in the terrestrial isopod Armadillidium vulgare
Source: Sci Rep. 2018 May 3;8:6998. doi: 10.1038/s41598-018-25450-4 (PMC5934373; doi:10.1038/s41598-018-25450-4)
Supplement: Supplementary file 3 — Supplementary information Figure S1 [file 41598_2018_25450_MOESM3_ESM.zip › Supplementary_Figure_S1.html]

 
RGL model


You must enable Javascript to view this page properly.

  
Drag mouse to rotate model. Use mouse wheel or middle button
to zoom it.

---

  
Object written from rgl 0.99.9 by writeWebGL.
